# Study on Kinetics of Transformation in Medium Carbon Steel Bainite at Different Isothermal Temperatures

**DOI:** 10.3390/ma14112721

**Published:** 2021-05-21

**Authors:** Wei Pei, Wei Liu, Yue Zhang, Rongjian Qie, Aimin Zhao

**Affiliations:** Collaborative Innovation Center of Steel Technology, University of Science and Technology Beijing, Beijing 100083, China; peiwei@xs.ustb.edu.cn (W.P.); b20160467@xs.ustb.edu.cn (W.L.); b20180502@xs.ustb.edu.cn (Y.Z.); qierongjian@xs.ustb.edu.cn (R.Q.)

**Keywords:** ultra-fine carbide-free bainitic steel, phase transition kinetics

## Abstract

Ultra-fine carbide-free bainitic (UCFB) steel, also known as nano-bainite (NB) steel, is composed of bainitic ferrite laths with nanoscale thickness and carbon-rich film-like retained austenite located between laths. The bainite transformation kinetic model can accurately describe the bainite transformation kinetics in conventional austempering (CA) processes based on the shear mechanism combined with the dilatometer test. UCFB steels with medium and high carbon composition are designed in this work to systematically study the transformation kinetics of bainite, and the evolution of its microstructure and properties, and reveal the influence of heat treatment processes on the microstructure and properties the UCFB steels. The results show that the activation energy for BF nucleation decreases during the CA process and isothermal transformation temperature decreases. The bainite transformation is first nucleated at the grain boundaries, and then nucleated at the newly formed bainitic ferrite/austenite interface.

## 1. Introduction

Bainitic phase transformation is an intermediate transition in the process of super-cooled austenite transformation. After holding the austenitized steel at a temperature slightly above the martensitic transformation temperature for a period of time, Bhadeshia [1] obtained a steel with a metallographic organization of nano-sized slatted bainitic ferrite and interslatted carbon-rich austenitic films. This steel, with high carbon and silicon content, has been widely noted for its excellent mechanical properties after heat treatment. Low-carbon bainitic steels that can be used to produce automotive parts have been researched extensively. The medium carbon bainitic steels have higher strength but less toughness than the low carbon bainitic steels. They can be used to produce wear-resistant parts for mines, railway rails, bearings and so on. Li [2] used bainitic steel, with a composition that ranged from 0.45% to 0.65% C, 2.00% to 4.00% Mn, 0.20% to 2.00% Si, to manufacture wear-resistant liners for mining machines that have more than double the service life under the same conditions than those made of Hadfield steel. With less hardness than martensitic steel, bainitic steel bearings show better wear resistance than martensitic steel bearings due to better toughness [3,4]. The increased toughness will further improve the wear resistance of the bearing [5]. 

The austenite that remains in the steel without transformation will have an important influence on the properties of the steel. The residual austenite in carbide-free bainite will yield first when receiving a load and then undergo a martensitic or bainitic phase transformation, which facilitates stress relief and can inhibit crack expansion under certain load conditions [6]. The volume and morphology of residual austenite in bainite can have a serious impact on the properties. Residual austenite with larger dimensions undergoes martensitic transformation in the early stages of the deformation process, while residual austenite distributed at grain boundaries with smaller size is more stable [7,8]. Bainitic transformation usually takes longer compared with martensitic transformation, and the time required for bainitic transformation of high-carbon steels is longer, even up to hundreds of hours [9]. By studying the kinetics of bainite phase transformation in Fe-2.98Cr-0.2Mn-0.38C (wt.%) steel, Kelly et al. [10] found that the initial bainite phase transformation is rapid, but then the phase transformation gradually stops. A higher isothermal transformation temperature will result in a faster bainite transformation rate, but less austenite will be converted to bainite. Pre-deformation can provide conditions for bainite nucleation and shorten the gestation time for bainite formation [11,12], but it can also reduce the growth rate of bainite and slow down the overall transformation rate [13]. The martensite produced by pre-phase transformation can also provide an interface for bainite nucleation and accelerate the process of bainite transformation [14,15]. In this article, the UCFB steels with medium carbon composition were designed to study the transformation kinetics of bainite and the evolution of its microstructure.

## 2. Materials and Methods

The steel investigated was medium-carbon-rich silicon steel with the chemical composition: 0.50C-2.56Si-1.07Mn-2.30Cr-0.0016Nb-0.0052B (wt.%). The preparation technology and phase transformation temperatures (Ac1, Ac3, Ms) of investigated steel are described in literature [16,17]. Ac1 and Ac3 were measured as 774 and 864 °C at a heating rate of 200 °C per h. Ms were measured as 187 °C at a cooling rate of 20 °C per s.

**Figure 1:**
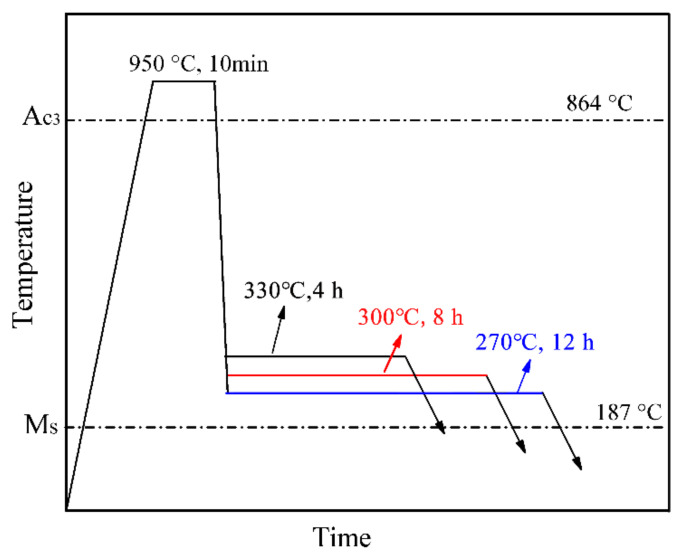
Schematic diagram of heat treatments used to understand bainite formation kinetics.

The conventional austempering experiments were conducted using DIL 805A, as shown in Figure 1. The conventional austempering consisted of austenitization at 950 °C for 10 min, followed by cooling to 330, 300 and 270 °C, and the corresponding hold for 4, 8 and 12 h, hereinafter referred to as CA-330, CA-300 and CA-270.

The microstructure morphology and phase distribution were characterized with a ZEISS ULTRA 55-type field emission scanning electron microscope (SEM) operating at 20 kV. Metallographic samples for SEM were ground, mechanically polished and etched with 2 vol. % nital. Transmission electron microscopy (TEM) was carried out on a Tecnai G2 F30 S-TWIN operated at 200 KV. The TEM samples were sliced into 300 μm from cylindrical samples. These were ground down to 50 μm, and then followed by double-jet thinning in the electrolyte of 5% perchloric acid and 95% glacial acetic acid, as the voltage was 25 V, the current was about 25 mA, and the temperature was 30 °C.

The volume fraction of retained austenite (RA) was measured by X-ray diffraction (XRD), operating at 40KV and a current of 150 mA with Cu-Kα radiation. The 2θ scanning angles were varied from 45 to 95 with a stepping angle of 0.05° and a counting time of 1.2 s per step using 10 × 10 mm^2^ specimens. The volume fraction of retained austenite was calculated by measuring the integrated intensities of the (200), (220) and (311) austenite peaks, and comparing them with (200), (211) ferrite peaks [18].

## 3. Results and Discussion

### 3.1. Subsection

**Figure 2:**
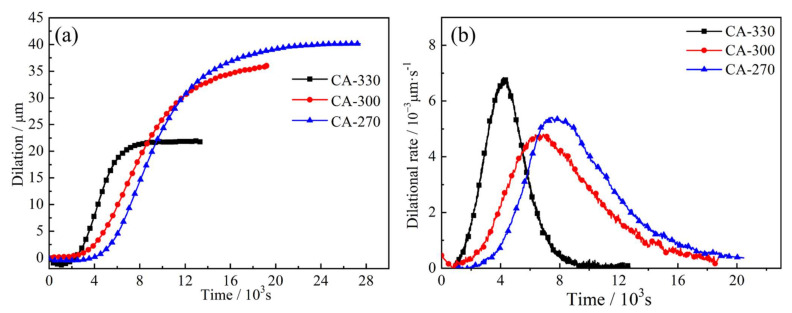
The dilation curves (**a**) and dilatational rate curves (**b**) of samples under CA process.

Figure 2 shows the dilation curves and dilatational rate curves of samples under the CA process. The straightness in the early stages and the end stages of the dilation curves indicates that the sample’s bainite transformation is in the incubation period and the completion stage. Data on thermal expansion of CA-330 and CA-270 are described in the literature [16,17]. The incubation period time and complete transition time of bainite are 1364 and 19,080 s for CA-270. As the temperature increases, the time required for both incubation and complete transformation becomes shorter. The relevant transition times and transition rates are listed in the Table 1.

### 3.2. Microstructure Characterizations

**Figure 3:**
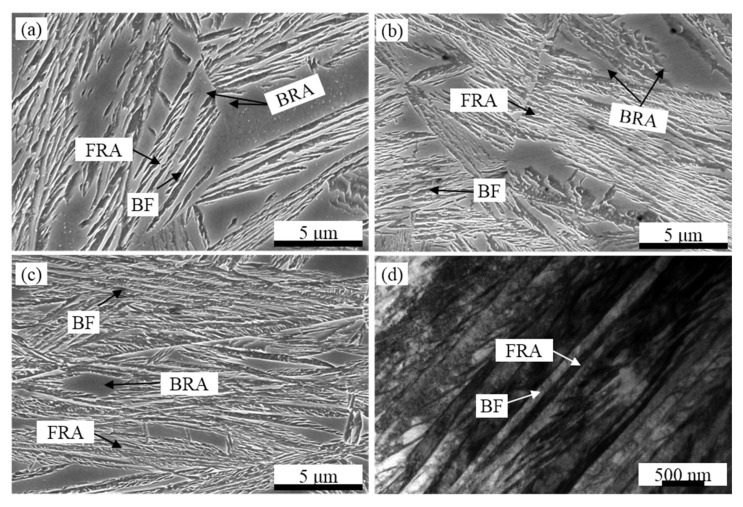
SEM (**a**–**c**) and TEM (**d**) microstructures of specimens austempered at various temperatures. FRA is film retained austenite; BRA is blocky retained austenite; BF is bainitic ferrite. (**a**) CA-330, (**b**) CA-300, (**c**,**d**) CA-270.

The typical microstructures of specimens are composed of bainitic ferrite (BF) and retained austenite (RA) under the CA process, as shown in Figure 3. According to the morphology of RA, it can be divided into the film retained austenite (FRA) and blocky retained austenite (BRA), as shown in Figure 3a–c. The TEM micrographs of the CA-270 sample are shown in Figure 3d. According to the formula [19], the BF thickness of CA-270 sample is 66 ± 18 nm.

**Figure 4:**
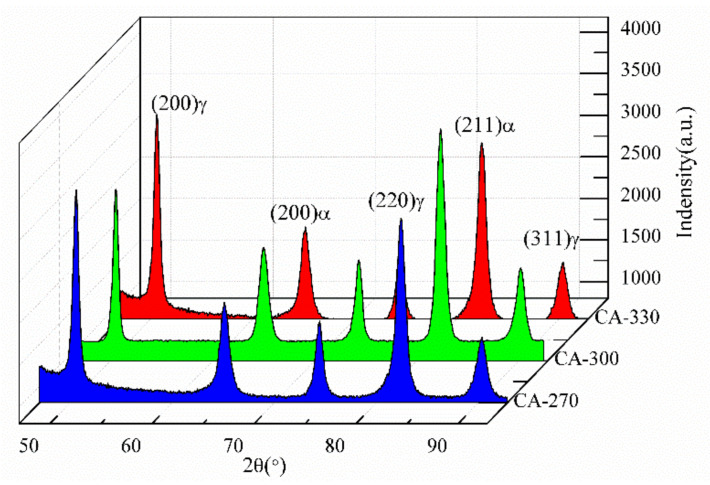
XRD patterns for samples isothermally treated at different temperatures.

The XRD patterns of the experimental steel at different temperatures are shown in Figure 4. The structure comprises BCC (BF) and FCC (RA) at different temperatures, and no carbides are generated. The content of RA in CA-330, CA-300 and CA-270 samples is 32.5%, 28.6% and 24.8%, respectively.

### 3.3. Bainite Transformation Kinetics during CA Process

#### 3.3.1. Fraction of Bainite Transformation

**Figure 5:**
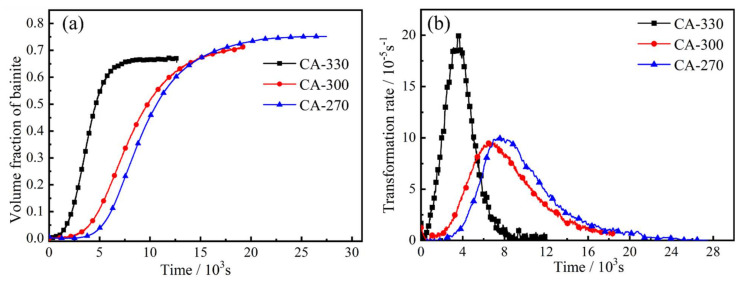
(**a**) Volume fraction of bainite, (**b**) transformation rate as a function of isothermal holding time at CA process.

Dilatometry data and XRD data can obtain the relationship between the volume fraction of bainite and isothermal holding time. The instantaneity volume fraction of bainite can be expressed by the following formula:

(1) $$f_{b}\left(t\right)=f_{b}\times \frac{L\left(t\right)-L_{0}}{L_{\infty }-L_{0}}$$

where *f_b_(t)* and *f_b_* are the instantaneity bainite volume fraction and the final bainite volume fraction, respectively. *L(t)*, *L*_0_ and *L*_∞_ stand for the instantaneity length, initial length and final length of the dilatometry sample. The variation in bainite volume fraction and bainite transformation rate with isothermal holding time is shown in Figure 5a,b, respectively. The variation trend of bainite transformation and bainite transformation rate with isothermal temperature is similar to that of expansion quantity. Therefore, the variation trend of bainite transformation and bainite transformation rate with temperature is not described here.

#### 3.3.2. Modeling of Bainite Transformation

In this work, the theoretical basis of predicting kinetics of bainite transformation in the CA process is the concept of the displacive theory of bainite transformation. According to the displacive theory of bainite transformation, bainite transformation occurs under the condition that both of the following conditions are met [20]:

(2) $$∆G_{m}{<G}_{N};\;\mathrm{where}\;∆G_{m}{=G}_{m}^{\alpha }{\;-\;G}_{m}^{\gamma }$$

(3) $$∆G^{\gamma \to \alpha }{<-G}_{\mathrm{SB}};\;\mathrm{where}\;∆G^{\gamma \to \alpha }{=G}^{\alpha }{\;-\;G}^{\gamma }$$

where ΔG_m_ is the maximum driving force for nucleation. $G_{m}^{\alpha }$ and $G_{m}^{\gamma }$ stand for the ferrite free energy and austenite free energy, respectively. G_N_ is the universal nucleation function [20]. $∆G^{\gamma \to \alpha }$ is the change in free energy in bainite transformation. $G^{\alpha }$ and $G^{\gamma }$ give the ferrite free energy and austenite free energy, respectively, when both the composition of ferrite and austenite is equal to the composition of interest. $G_{\mathrm{SB}}$ is the stored energy of bainite, usually considered to be 400 J mol^−1^ [20]. The temperature at which $∆G_{m}{=G}_{N}$ and $∆G^{\gamma \to \alpha }{=-G}_{\mathrm{SB}}$ are called *T_h_* and $T_{0}^{\prime }$ temperature, respectively [21].

The bainite shear theory believes that bainite nucleates and grows at the interface. At the beginning of bainite transformation, bainite ferrite nucleates at prior austenite/austenite interface, that is, grain boundary nucleation. Once the bainite ferrite is formed, the bainite will nucleate at the tip of interface newly formed bainite/austenite; that is, autocatalytic nucleation. The microstructure of the experimental steel treated by the CA process is composed of bainitic ferrite and retained austenite, and the interfaces present in the tissue are the pristine austenite interface and the bainitic ferrite/ferrite interface. According to Bohemen and Sietsma’s transformation model and the model improved by Ashwath, the overall bainite nucleation rate of the experimental steel under the CA process can therefore be expressed as the sum of the grain boundary nucleation rate and the autocatalytic nucleation rate.

The bainite shear theory states that bainite is nucleated and grows at the interface between different phases. The interfaces present in the specimens are the original austenite interface and the bainitic ferrite/ferritic interface. At the beginning of bainite transformation, bainitic ferrite is first nucleated at the pristine austenite interface (grain boundary nucleation). Once bainitic ferrite is generated, the bainitic ferrite nucleates at the tip of the freshly formed bainitic ferrite/austenite interface (autocatalytic nucleation). The overall bainite nucleation rate of this experimental steel under the CA process can be expressed as the sum of the grain boundary nucleation rate and the autocatalytic nucleation rate. The formula is expressed as:

(4) $$\frac{df}{dt}={\left(\frac{df}{dt}\right)}_{G}+{\left(\frac{df}{dt}\right)}_{A}=\frac{kT}{h}\frac{Z\delta }{d}mf_{\gamma }\left(T_{h}-T\right)\left[e^{\frac{-Q_{G}^{*}}{RT}}+f_{b}e^{\frac{-Q_{A}^{*}}{RT}}\right]\;\;\;\;\;\;\;\;\;\;\;\;\;\;\;\;\;\;\;\;\;=\frac{kT}{h}\frac{Z\delta }{d}m\left(T_{h}-T\right)\left(1-f\right)\frac{T_{0}^{\prime }-T}{T_{0\bar{X}}^{\prime }-T}\left[1+f_{b}e^{\frac{∆Q^{*}}{RT}}\right]\left(e^{\frac{-Q_{G}^{*}}{RT}}\right)$$

where: (*df/dt*)*_G_* and (*df/dt*)*_A_* are grain-boundary nucleation and autocatalytic nucleation, respectively; *k* for Boltzmann constant, 1.38 × 10^−23^ J·*K*^−1^; *h* for Planck constant, 6.626 × 10^−34^ J·s; *Z* for geometrical factor, 6; *δ* for the effective thickness of the austenite grain boundary, 1 nm; *d* for the prior austenite grain diameter; *m* for the kinetics parameter giving an account of the relationship between martensite nucleation number and the degree of undercooling in *K*^−1^; *f_r_* for volume fraction of remaining available austenite; *f* for volume fraction of bainite; *R* for the molar gas constant, 8.314 J·mol^−1^·*K*^−1^; $Q_{G}^{*}$ and $Q_{A}^{*}$ for the activation energy of bainite nucleation at grain boundary and autocatalytic nucleation, respectively; ΔQ* for the difference in activation energy between grain boundary nucleation and autocatalytic nucleation; *T_h_* for the maximum temperature that the bainite nucleus can develop;$\;T_{0}^{\prime }$ for the highest temperature for which the diffusionless growth of bainite can take place; and the parameters (*T_h_*, $T_{0}^{\prime }$ and $Q_{G}^{*}$) vary linearly with an increase in carbon enrichment of austenite, and are described in the literature [22].

(5) $$T_{h}=T_{h\bar{X}}-C_{1}\frac{f\left(\bar{X\;}{-\;X}_{b}\right)}{1\;-\;f}$$

(6) $$T_{0}^{\prime }=\;T_{0\bar{X}}^{\prime }\;-C_{2}\frac{f\left(\bar{X}\;-\;X_{b}\right)}{\left(1\;-\;f\right)}$$

(7) $$Q_{G}^{*}=Q_{G\bar{X}}^{*}+K_{Г}C_{1}\frac{f\left(\bar{X\;}\;-\;X_{b}\right)}{1\;-\;f}$$

where: $T_{h\bar{X}}$, $\;T_{0\bar{X}}^{\prime }$ and $Q_{G\bar{X}}^{*}$ are the *T_h_* temperature, $T_{0}^{’}$ temperature and activation energy for grain-boundary nucleation at start of the transformation; *C*_1_ (*C*_2_) is the constant between $T_{h}$ ($T_{0}^{\prime }$) and carbon content; and $X_{b}$ and $\bar{X}$ are the carbon content in bainite and bulk carbon content.

The values of various parameters in the model are shown in Table 2. The parameters of $T_{h\bar{X}}$, $\;T_{0\bar{X}}^{\prime }$, *C*_1_ and *C*_2_ were calculated by a computer program, MUCG 83. *K_Г_* and m are calculated by the empirical formula described in papers [23,24,25]. The grain size of prior autenite was simulated by JMatPro software.

**Figure 6:**
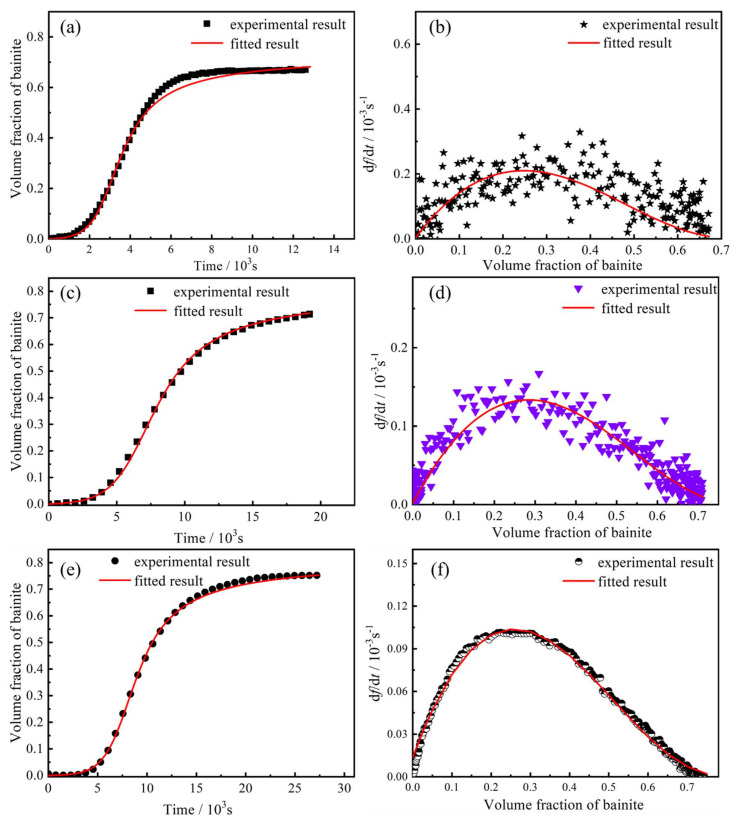
The calculated values and experimental values of CA-330 sample, CA-300 sample and CA-270 sample. (**a**,**c**,**e**) volume fraction of bainite as a function of isothermal holding time; (**b**,**d**,**f**) transformation rate as a function of volume fraction of bainite.

The calculated curves have a very high fitting degree with the experimental curves, especially in the initial stage of bainite transformation, as shown in Figure 6. Figure 6a,c,e depicts the comparison between the fitted values and experimental values of bainite volume fraction as a function of isothermal holding time. In the initial stage of bainite transformation, the nucleation position of bainite is mainly prior austenite boundary. Therefore, the fitted value of $Q_{G\bar{X}}^{*}$ is very reliable. With bainite transformation, bainite gradually increased; grain-boundary nucleation and autocatalytic nucleation are the main types of bainite. The reasonable degree of this part is also very high. When the bainite transformation enters the later stage, due to the increase in bainite ferrite and the depletion of grain boundary nucleation position, the bainite transformation mainly takes the form of autocatalytic nucleation. The fitting results show that the reasonable degree of CA-300 and CA-270 is relatively high, indicating that the relevant result of autocatalytic activation energy is highly reliable. However, the reasonable degree of CA-330 is quite different in the later transformation stage, indicating that autocatalytic activation energy’s relevant result has a large deviation. Figure 6b,d,f is a fitting comparison diagram of the transformation rate. It can be seen that the fitting results of the bainite transformation rate have the same trend as the experimental results. The fitting results in the latter part of the transformation rate fitting results show that the applicable rate is significantly slower than the experimental results, as shown in Figure 6b, which indicates that the autocatalytic activation energy is larger in the late stage of the process.

**Figure 7:**
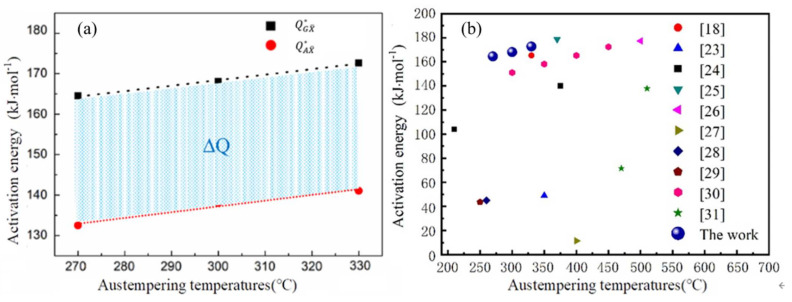
(**a**) The value of $Q_{G\bar{X}}^{*}$ and $Q_{A\bar{X}}^{*}$ as a function of undercooling; (**b**) activation energy in this paper value compared with other research results.

As shown in Figure 7a, the model’s value of $Q_{G\bar{X}}^{*}\;$ and ΔQ* at different austempering temperatures are fitted. The value of $Q_{A\bar{X}}^{*}$ at different isothermal temperatures is also calculated. The $Q_{G\bar{X}}^{*}$ and $Q_{A\bar{X}}^{*}$ show a linear decline trend with the austempering temperature. The results are the same as those reported in the literature [22,26], which indicates that the fitting results in this paper are credible. It should be noted that the activation energy is not divided into grain boundary activation energy and autocatalytic activation energy, except in the literature [22,27]. In Figure 7a, the blue area between the black dotted line and the red dotted line represents the difference between the initial grain boundary activation energy and the autocatalytic activation energy, ranging from 30 to 32 kJ/mol. Figure 7b describes bainite’s activation energy of different experimental steels, which ranges from 11 to 180 kJ/mol [22,27,28,29,30,31,32,33,34,35]. The activation energy of bainite isothermal transformation is all within the range, which indicates that the fitting results are reliable.

**Figure 8:**
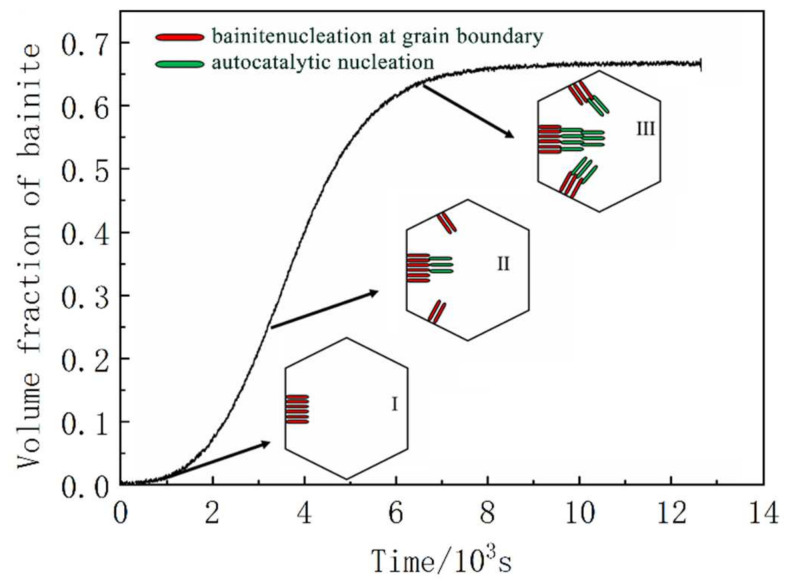
Schematic diagram of bainite nucleation under CA process.

The schematic diagram of bainite nucleation and growth under the CA process can be summarized, as shown in Figure 8. At the initial stage of bainite transformation (stage Ι), the bainite transformation amount is very small. The microstructure interfaces are mainly austenite/austenite interfaces, bainite nucleation at the grain boundary. The number of bainite ferrite/austenite interfaces increases with the increase in bainite content. Bainite can nucleate at the newly formed bainite ferrite/austenite interface. At this stage (stage ΙΙ), bainite nucleates at the austenite/austenite interface and the ferrite/austenite interface. As the bainite transformation enters the later stage (stage ΙΙΙ), the prior austenite grain boundary’s nucleation position is exhausted, and the available volume fraction of austenite decreases, the nucleation rate of bainite decreases. It is mainly dominated by the bainite ferrite/austenite interface, namely autocatalysis nucleation.

## 4. Conclusions

Isothermal bainite transformation was carried out at different temperatures in this experiment. The results show that a higher content of residual austenite is obtained by isothermal transformation at higher temperatures, with lower carbon content in the residual austenite. The residual austenite content in the steel after isothermal phase transformation at 330, 300 and 270 °C was 32.5%, 28.6% and 24.8%, respectively. In addition to this, less time is required for the bainite transformation to reach its maximum transforming rate when the isothermal transformation is higher. In contrast, the bainite ferrite thickness, film residual austenite thickness, and body residual austenite size become smaller when the bainite transformation temperature is lower. In contrast, the bainite ferrite thickness, film residual austenite thickness and body residual austenite size become smaller when the bainite transformation temperature is lower. The activation energy for nucleation at the grain boundaries is higher than that for nucleation at the bainitic ferrite/austenite interface.

## Figures and Tables

**Table 1:** Bainite phase transformation parameters at different isothermal temperatures.

	CA-330	CA-300	CA-270
Incubation Period Time/s	804	1364	3000
Complete Transition Time/s	10,962	19,080	26,980
Maximum Transition Speed time/s	4217	6600	7603
Maximum Transformation Rate/μm·s^−1^	0.0067	0.0047	0.0054

**Table 2:** The calculated values of various parameters in the model.

$T_{h\bar{X}}/K$	*C*_1_/*K*	$T_{0\bar{X}}^{\prime }/K$	*C*_2_/*K*	*K_Г_*/J·mol^−1^·*K*^−1^	*m*/*K*^−1^	*d*/μm
680.6	100.0	776.5	168.8	110.1	0.015	34

## Data Availability

This study did not report any data.
